# Enhancing dermatological diagnosis for differentiating actinic from seborrheic keratosis using deep learning model

**DOI:** 10.3389/fmed.2025.1654813

**Published:** 2025-10-02

**Authors:** Ying-Ying Ren, Li-Hong Mei, Xiang-Dong Liu, Zhe Quan, Gao Yang

**Affiliations:** ^1^Department of Dermatology, Jinshan Hospital, Fudan University, Shanghai, China; ^2^Department of Dermatology, Shanghai Sixth People's Hospital, Jiaotong University, Shanghai, China; ^3^Department of Dermatology, Shanghai United Family Pudong Hospital, Shanghai, China

**Keywords:** actinic keratosis, seborrheic keratosis, deep learning, dermatologist assistance, computer-aided diagnosis

## Abstract

**Background:**

Differentiating Actinic keratosis (AK) from Seborrheic keratosis (SK) can be challenging for dermatologists due to their visual similarities. This multi-center prospective study aims to investigate the efficacy of deep learning (DL) model in assisting dermatologists in accurately classifying AK from SK lesions.

**Methods:**

A contrastive language-image pre-training (CLIP) model with ViT-B/16 architecture was trained on an dataset of 2,307 patients and validated in three separate datasets of 386 (from Center A), 196 patients (from Center B and C) and 215 patients (from DermNet). Two dermatologists classified the lesions separately. Then they were showed the model’s predictions and were requested to reclassify the results if needed. Area under the receiver operating characteristic (ROC) curve (AUC) was used to evaluate the diagnostic performances of the DL model and the dermatologists before and after reclassification. The change in the dermatologists’ classification decisions was also analyzed by net reclassification index (NRI) and total integrated discrimination index (IDI).

**Results:**

The model’s diagnostic performance in the training cohort and validation cohort 1, 2 and 3 showed an AUC of 0.85, 0.89, 0.84, and 0.89. For dermatologist 1, the diagnostic performance improved from 0.77 to 0.80 in the test cohort with NRI and IDI of 0.10 (*p* = 0.006) and 0.14 (*p* < 0.001). For dermatologist 2, the diagnostic performance increased from 0.69 to 0.79 with NRI and IDI of 0.19 (*p* < 0.001) and 0.27 (*p* < 0.001).

**Conclusion:**

The DL model significantly improves dermatologists’ accuracy in differentiating AK from SK, especially for less experienced ones. The DL model has the potential to reduce diagnostic subjectivity, aid early detection of precancerous lesions, and transform dermatological diagnostic and therapeutic practices.

## Introduction

Skin cancer represents a significant global health burden (1). Actinic keratosis (AK) is a prevalent precancerous lesion that develops as a consequence of long-term sun exposure (2). Accurate diagnosis of AK is critical for ensuring effective treatment and assessing therapeutic outcomes. In contrast, seborrheic keratosis (SK) is the skin growth of keratinocytes, which is one of the most common benign lesions (3). AK is a precancerous lesion with malignant potential, while SK is benign and typically requires no treatment. Despite their distinct prognostic implications, AK and SK often present with overlapping clinical and dermoscopic features. Accurate differentiation AK from SK is challenging, even for experienced dermatologists.

Histopathological examination remains the gold standard, it is invasive, time-consuming, and impractical for routine screening. Traditional diagnosis of AK and SK relies on subjective visual inspection. Dermatologists’ experience and interpretation can influence their evaluation, potentially leading to inter-observer variability and missed diagnoses (4). Additionally, visual inspection alone may not capture subtle features crucial for differentiating AK from SK (5). These limitations can result in unnecessary intervention for SK or delayed treatment for AK, which can progress to squamous cell carcinoma if left untreated (6). Thus, there is a critical need for more objective and accurate diagnostic tools to improve dermatological diagnosis of AK and SK.

Deep learning (DL) is a powerful sub-field of artificial intelligence, which offers a promising solution for image analysis in healthcare (7). DL models have demonstrated remarkable success in various medical image classification tasks, including skin lesion analysis (8). Previous study showed that the DL model could achieve dermatologist-level accuracy in classifying skin cancers from dermatological images (9). Wang et al. proposed a DL model to improve automatic medical image classification for malignant skin lesions, which showed good performance and potential for further development (10). Zhang et al. used a DL model to differentiate scalp psoriasis from seborrheic dermatitis, which outperforming dermatologists in accuracy. The model boosted the diagnostic skills of less experienced dermatologist with high efficiency (11). We also previously assessed a DL model’s effectiveness in aiding dermatologists to classify basal cell carcinoma from SK, finding that the DL model significantly improved diagnostic accuracy and reduced misdiagnoses (12). Reddy et al. developed a DL model to diagnosis AK and SK. The findings emphasize the DL model’s ability in accurate distinguish AK from SK (13). However, the role of DL models in improving dermatological diagnosis and treatment decisions for AK and SK has not been fully investigated or validated across different datasets.

We assumed that DL models could be used to classify AK and SK and further improve the dermatologists’ diagnostic performance. In this study, we developed a DL model specifically designed for AK and SK classification and validated it on different datasets. We further evaluate the usefulness of this DL model in improving diagnostic accuracy of the dermatologists in differentiating AK from SK.

## Materials and methods

### Ethics statement

This study was conducted in accordance with the Declaration of Helsinki. This study was reviewed and approved by the Institutional Review Board of Jinshan Hospital (JIEC 2023-S85). Written informed consent was obtained from all participants prior to enrollment for publication of any potentially identifiable data or images.

### Study design

This prospective study aimed to assess the effectiveness of DL model in classifying AK and SK. Participants with histopathologically confirmed AK or SK were included. The datasets included: a cohort of 2,307 patients from the international skin imaging collaboration dataset (ISIC, https://www.isic-archive.com), a cohort of 386 patients from Center A, a cohort of 196 patients from Center B and Center C, and a cohort of 215 patients from DermNet (https://dermnetnz.org/images).

### Datasets and data split

From November 1, 2023, to April 1, 2024, adult patients undergoing surgical resection for skin neoplasm were enrolled from three centers (Center A, Center B and Center C). The inclusion criteria were as follows: (1) Histopathologically confirmed AK or SK; (2) Age ≥ 18 years. Exclusion criteria included: (1) Presence of systemic infection; (2) Incomplete clinical data; (3) Images with motion blur or artifacts.

The data from ISIC with histopathologically confirmed AK and SK were used as a training cohort for training the DL model. The data from Center A was used as a validation dataset1; the data from Center B and Center C were combined and used as a validation dataset2; the data from DermNet (histopathologically confirmed clinical photographs) was used as a validation dataset3. These dataset were used for validating the DL model.

### Image Preprocessing

Image preprocessing steps are consistent with what we previously reported (12). Briefly, the images were captured by dermoscopy or devices with a minimum camera resolution of 12 megapixels. Adequate natural daylight or bright artificial light was used for clear visibility of the skin lesions. All images were resized to a standard size suitable for the input layer of DL models and converted into tensor format. The preprocessing stage included data augmentation operations such as random cropping, rotation, flipping, and color transformations. The normalization process was performed by subtracting the mean value of the entire dataset and dividing by the standard deviation to normalize pixel values to a standard range.

### DL model architecture

The DL model was based on a contrastive language-image pre-training (CLIP) model with ViT-B/16 architecture (14, 15). Images were first divided into a set of fixed-size patches, each patch was then flattened and embedded into a vector. These vectors were then passed as input to the Transformer encoder to model the image globally. Finally, these representations went through several fully connected layers to produce the final classification or regression outputs. To benchmark CLIP-ViT against widely used CNNs like ResNet or EfficientNet, we also conducted a head-to-head evaluations of CLIP-ViT and conventional CNNs (ResNet-50 fine-tuned on the same data sets) to clarify transformer-based architectures’ advantages.

### Feature extraction

The input images were divided into fixed-size image patches, and each image patch was mapped to a low-dimensional space through a learnable linear projection, forming an embedding vector for the image patch. Position encodings were added to the embedding vectors of each image patch to represent the relative positional relationships between the image patches. A Transformer encoder was then employed to encode the sequence of embedding vectors, which included self-attention mechanisms and fully connected feed-forward networks to capture semantic information and contextual relationships between the image patches. A fixed-length vector representation was obtained through feature pooling, which was then projected through a fully connected layer to the same embedding space as the text features.

### Training parameters

The model was trained using the stochastic gradient descent (SGD) optimizer with momentum. The initial learning rate, momentum, and weight decay were set to 0.002, 0.9, and 0.005, respectively. We adopt the ViT-B/16 variant of the CLIP model and set the number of the learnable prompt vectors to 16. The number of training epochs is set to 100. The input images of the dataset were all resized to 224 × 224 pixels. To ensure reproducibility, we set the random seed to 0 and used a batch size of 32 to maintain training efficiency.

Cross-entropy loss was utilized to measure the disparity between the predicted results and the true labels. The outputs of the model were first processed through a softmax function to transform them into a probability distribution. Then, these probabilities were compared with the true labels to compute the cross-entropy loss between the model’s predictions and the true labels. For each sample, the cross-entropy loss was the negative log-likelihood of the predicted label at the corresponding position (16).

### Computational requirements and runtime

Our server was equipped with two NVIDIA RTX 4090 GPUs, each with 24GB of memory. The central processing unit (CPU) was an Intel Core i9-13900K, featuring 24 cores and 36 threads. 256 GB of DDR4 RAM was used for memory. Data storage was managed with a 1 TB SSD, enabling fast data read and write operations. The operating system was Ubuntu 20.04, and the software environment included tools and libraries such as PyTorch 1.10, CUDA 11.2, Anaconda 23.3.1, CUDA 12.0, cuDNN v8.8.1, PyTorch 1.13.1, and Python 3.7.16. For the public ISIC dataset, the total training time for 100 epochs was approximately 1.1 h, while the average inference time per instance was 20.50 milliseconds. During the inference phase, the average inference time per instance was 2.56 milliseconds, with a throughput of 390 samples per second.

### Evaluation metrics

Attention mechanisms were employed to visualize the model’s regions of interest during image recognition. Specifically, attention maps were generated to illustrate the model’s focus on different regions of the images. The attention maps revealed the image features that the model primarily relied on for predictions, such as color, shape, and texture. Grad-CAM was utilized to highlight the regions in the images considered by the model as crucial for predicting the corresponding labels. The area under the receiver operating characteristic (ROC) curves (AUC) were used to evaluate the clinical application of the DL model in assisting dermatologists.

### DL model in assisting dermatologists

First, two dermatologists (dermatologist 1 with 15 years and dermatologist 2 with 3 years of experience, both blinded to histopathological data) reviewed the images to identify AK or SK. Second, each dermatologist was shown the classification result of the DL model. The dermatologists were allowed to reclassify the diagnosis if needed according to the DL results. Any changes of the dermatologist in reclassification were recorded. Net reclassification index (NRI) and total integrated discrimination index (IDI) were calculated to compare the discrimination performances of the dermatologists before and after considering the DL model’s results (17).

### Statistical analysis

Statistical analysis was performed using R software (version 4.3.2; https://www.r-project.org/). Data normality and homogeneity of variance were assessed using appropriate tests. For continuous variables, independent-samples t-tests (met the assumptions of normality) or non-parametric Mann–Whitney U test (not met the assumptions of normality) was performed. Categorical variables were compared using the chi-squared test or Fisher’s exact test. A *p*-value less than 0.05 was considered statistically significant.

## Results

### Datasets

**Figure 1:**
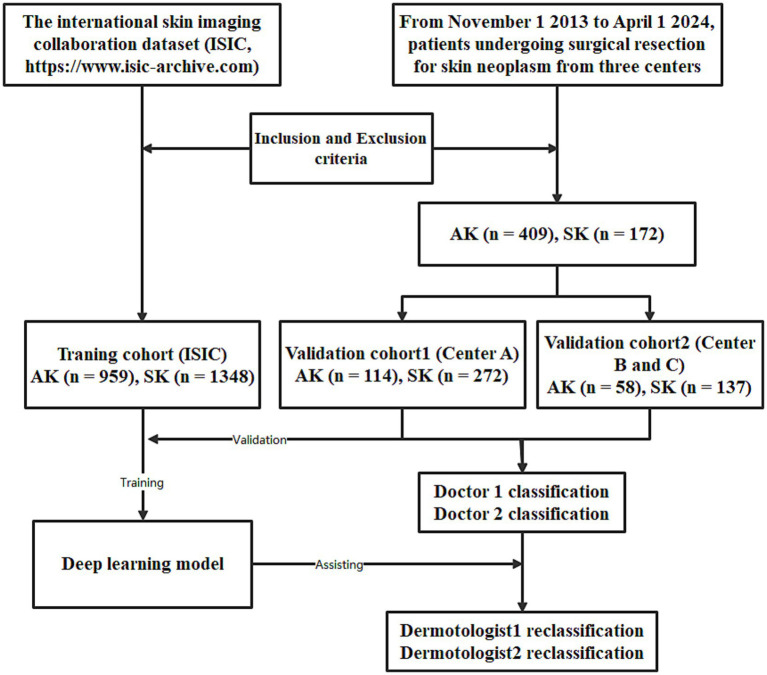
The workflow of the deep learning (DL) model developed for the classification of actinic keratosis (AK) and seborrheic keratosis (SK).

**Figure 2:**
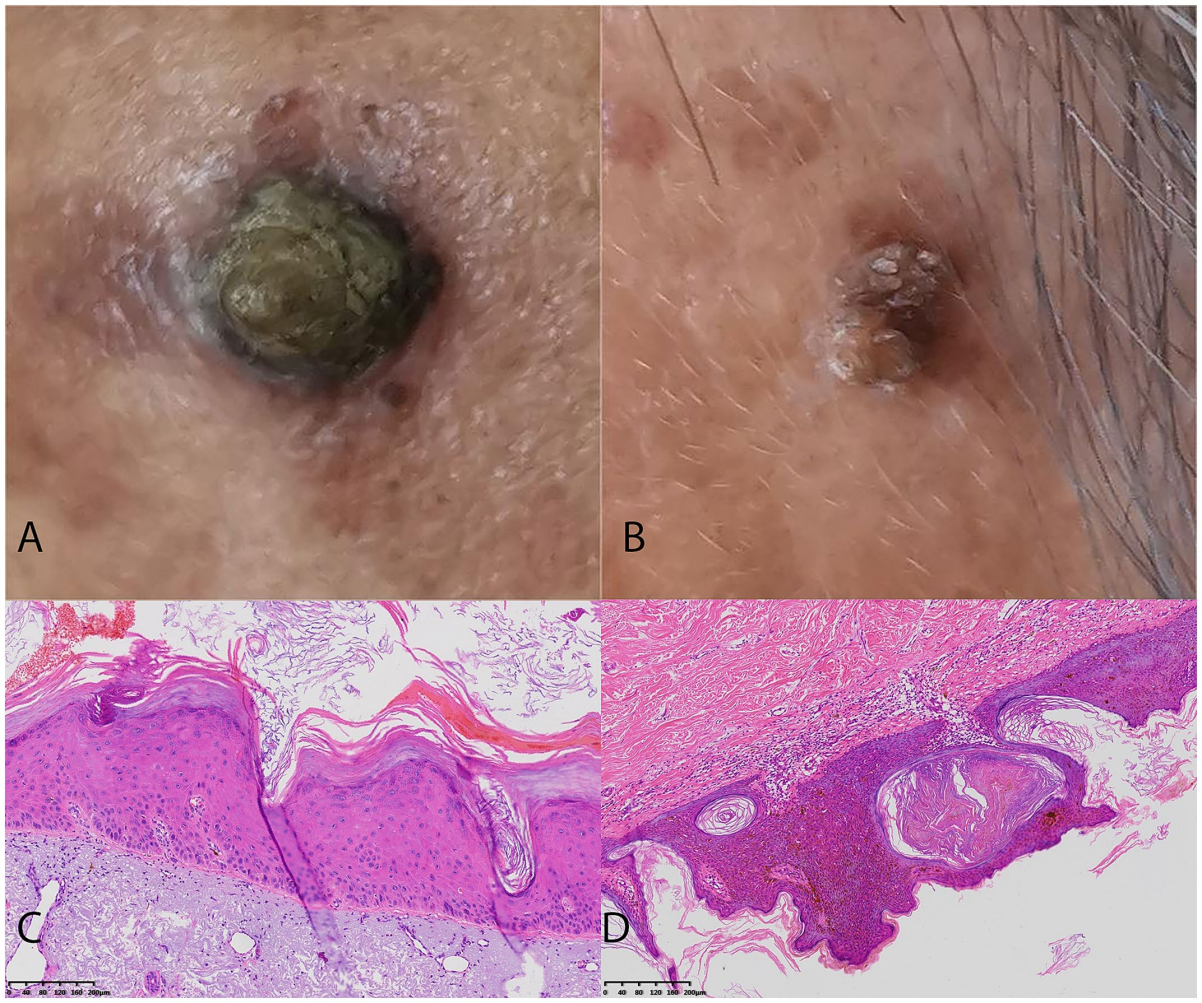
Two challenging case examples of AK and SK classification. **(A)** An example of a uncorrectly classified lesion of pigmented AK lesion by Dermatologist 2, but the model’s prediction aligns with the histopathological diagnosis. The Dermatologist 2 reclassified it to AK correctly with the assistance of DL model. **(B)** An example of a uncorrectly classified SK lesion with overlapping features mimicking AK by Dermatologist 2, but the model’s prediction aligns with the histopathological diagnosis. The Dermatologist 2 reclassified it to SK correctly with the assistance of DL model. **(C)** The histopathological findings of the AK case with the presence of atypical keratinocytes in the epidermis, parakeratosis, and an irregular, thickened stratum corneum. **(D)** The histopathological findings of the SK case with the presence of acanthosis, hyperkeratosis, and horn cysts.

The training cohort (ISIC dataset) included a total of 2,307 patients (1,004 females and 1,303 males, aged 64 ± 13, ranged from 20 to 85), with 1,348 diagnosed with SK and 959 diagnosed with AK. The validation dataset1 included a total of 386 patients (211 females and 175 males, aged 60 ± 15, ranged from 21 to 95). The validation dataset2 included a total of 195 patients (138 females and 57 males, aged 59 ± 16, ranged from 23 to 91). The workflow of this study is shown in Figure 1. The clinical characteristics of patients in training and validation cohorts are shown in Table 1. Two case examples of AK and SK is shown in Figure 2.

**Table 1:** Clinical characteristics of patients with actinic keratosis (AK) and seborrheic keratosis (SK).

		Training cohort		Validation cohort1			Validation cohort2		Validation cohort3		
Parameters	SK (*N* = 1,348)	AK (*N* = 959)	*p*-value	SK (*N* = 272)	AK (*N* = 114)	*p*-value	SK (*N* = 137)	AK (*N* = 58)	*p*-value	SK (*N* = 50)	AK (*N* = 165)
Gender			<0.001			0.625			0.125		
Female	543 (40.3%)	461 (48.1%)		146 (53.7%)	65 (57.0%)		92 (67.2%)	46 (79.3%)		-	-
Male	805 (59.7%)	498 (51.9%)		126 (46.3%)	49 (43.0%)		45 (32.8%)	12 (20.7%)		-	-
Age	63.0 ± 14.0	66.5 ± 12.2	<0.001	55.8 ± 13.4	71.5 ± 11.9	<0.001	54.7 ± 14.2	69.2 ± 16.2	<0.001	-	-
CLIP model			<0.001			<0.001			<0.001		
SK	1,128 (83.7%)	123 (12.8%)		257 (94.5%)	18 (15.8%)		134 (97.8%)	17 (29.3%)		49 (98%)	32 (19.4%)
AK	220 (16.3%)	836 (87.2%)		15 (5.5%)	96 (84.2%)		3 (2.2%)	41 (70.7%)		1 (2%)	133 (80.6%)
Dermatologist 1						<0.001			<0.001		
SK	-	-		222 (81.6%)	26 (22.8%)		102 (74.5%)	12 (20.7%)		-	-
AK	-	-		50 (18.4%)	88 (77.2%)		35 (25.5%)	46 (79.3%)		-	-
Dermatologist 1 with DL						<0.001			<0.001		
SK	-	-		240 (88.2%)	12 (10.5%)		132 (96.4%)	13 (22.4%)		-	-
AK	-	-		32 (11.8%)	102 (89.5%)		5 (3.6%)	45 (77.6%)		-	-
Dermatologist 2						<0.001			<0.001		
SK	-	-		242 (89.0%)	39 (34.2%)		126 (92.0%)	31 (53.4%)		-	-
AK	-	-		30 (11.0%)	75 (65.8%)		11 (8.0%)	27 (46.6%)		-	-
Dermatologist 2 with DL						<0.001			<0.001		
SK	-	-		254 (93.4%)	38 (33.3%)		133 (97.1%)	23 (39.7%)		-	-
AK	-	-		18 (6.6%)	76 (66.7%)		4 (2.9%)	35 (60.3%)		-	-

### Model performance

The architecture of the DL model is shown in Supplementary Figure 1. For the training cohort (ISIC dataset), the model demonstrated an AUC of 0.85 with sensitivity of 0.87 and a specificity of 0.84, with PPV and NPV of 0.79 and 0.90, respectively.

For the validation cohort1 (Center A), the initial performance metrics of the model in the training cohort revealed an AUC of 0.89, indicating a moderate discrimination ability. The model demonstrated a sensitivity of 0.84 and a specificity of 0.94, with positive predictive value (PPV) and negative predictive value (NPV) of 0.86 and 0.93, respectively.

For the validation cohort2 (Center B and C), the model’s accuracy and discrimination capabilities were further affirmed, with an AUC of 0.84 showcasing an excellent ability to differentiate between AK and SK. The model achieved a sensitivity of 0.71 and a specificity of 0.98. The PPV and NPV were noted at 0.93 and 0.89, respectively (Table 2).

**Table 2:** Area under the curve (AUC) for model performance and dermatologist assessments.

Parameters	Models	AUC	95%CI	SPE	SEN	NPV	PPV
Training cohort	CLIP model	0.85	0.84–0.87	0.84	0.87	0.90	0.79
Validation cohort1	CLIP model	0.89	0.86–0.93	0.94	0.84	0.93	0.86
	Dermatologist 1	0.79	0.75–0.84	0.82	0.77	0.90	0.64
	Dermatologist 1 with CLIP	0.89	0.85–0.92	0.88	0.89	0.95	0.76
	Dermatologist 2	0.77	0.73–0.82	0.89	0.66	0.86	0.71
	Dermatologist 2 with CLIP	0.80	0.75–0.85	0.93	0.67	0.87	0.81
Validation cohort2	CLIP model	0.84	0.78–0.9	0.98	0.71	0.89	0.93
	Dermatologist 1	0.77	0.7–0.83	0.74	0.79	0.89	0.57
	Dermatologist 1 with CLIP	0.87	0.81–0.93	0.96	0.78	0.91	0.90
	Dermatologist 2	0.69	0.62–0.76	0.92	0.47	0.80	0.71
	Dermatologist 2 with CLIP	0.79	0.72–0.85	0.97	0.60	0.85	0.90
Validation cohort3	CLIP model	0.89	0.86–0.93	0.98	0.81	0.60	0.99

AUC, area under the curve; CLIP, contrastive language-image pre-training; PPV, positive predictive value; NPV, negative predictive value; SEN, sensitivity; SPE, specificity.

For the validation cohort3 (DermNet with 165 AK and 50 SK), the model achieved an AUC of 0.89 in differentiating AK from SK with a sensitivity of 0.81 and a specificity of 0.98. The PPV and NPV were of 0.99 and 0.60, respectively (Table 2). The comparison of the performance of resnet50 fine-tuned and CLIP on the same data sets is shown in Supplementary Table 1.

### The performance of the DL model in assisting dermatologists

**Figure 3:**
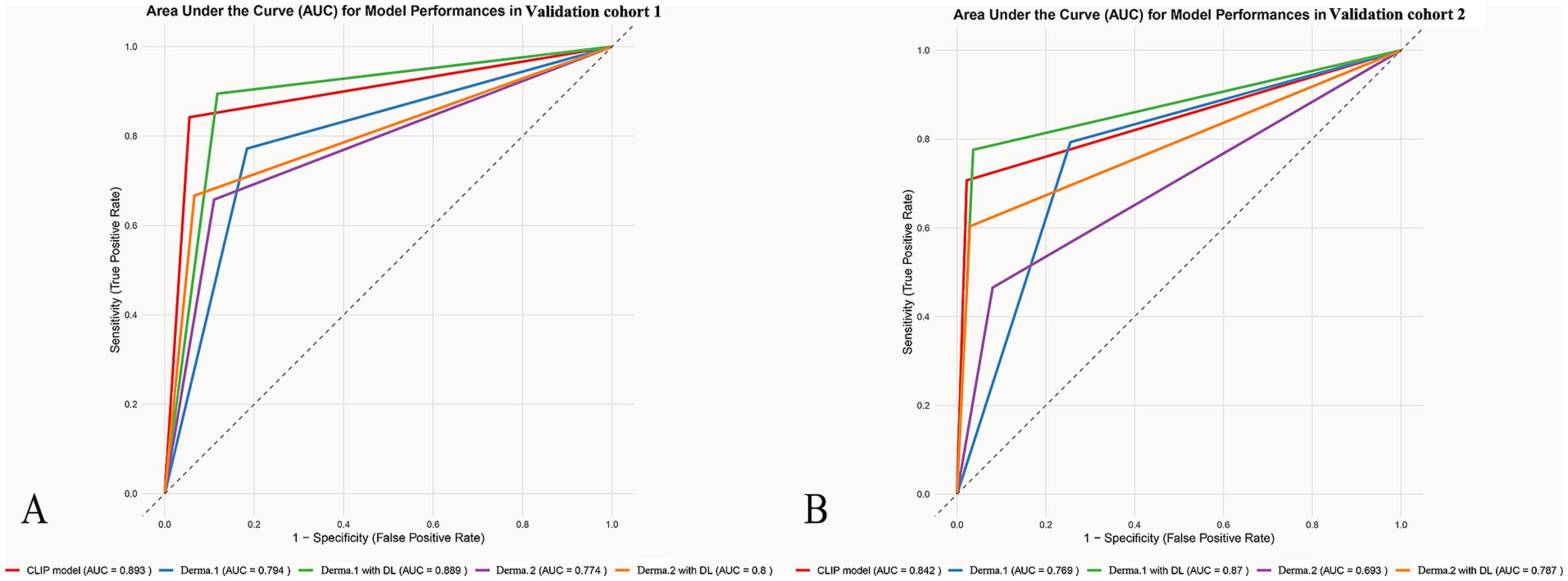
The ROCs for the different model’s classification of AK and SK. The ROC graphs provide a visual representation of the model’s discriminatory power compared to dermatologists with accuracy, sensitivity, and specificity in differentiating AK from SK across different datasets **(A)** validation cohort1 **(B)** validation cohort2.

Without the DL model’s assistance, dermatologist 1 achieved AUCs of 0.77 and 0.69 in diagnosing SK from AK with SEN, SPE, PPV and NPV of 0.66, 0.89, 0.71, and 0.86 and 0.47, 0.92, 0.71, and 0.80 for the validation cohort1 and 2. Dermatologist 2 achieved AUCs of 0.79 and 0.77 in diagnosing SK from AK with SEN, SPE, PPV and NPV of 0.77, 0.82, 0.64, and 0.90 and 0.86 and 0.79, 0.74, 0.57, and 0.89 for the validation cohort1 and 2 (Figure 3).

After giving the predict results of the model, the dermatologist 1 achieved AUCs of 0.80 and 0.80 in diagnosing SK from AK with SEN, SPE, PPV and NPV of 0.67, 0.93, 0.81, and 0.87 and 0.60, 0.97, 0.90 and 0.85 for the validation cohort1 and 2. The dermatologist 2 achieved AUCs of 0.89 and 0.87 in diagnosing SK from AK with SEN, SPE, PPV and NPV of 0.89, 0.88, 0.76, and 0.95 and 0.78, 0.96, 0.90, and0.91 for the validation cohort1 and 2.

**Figure 4:**
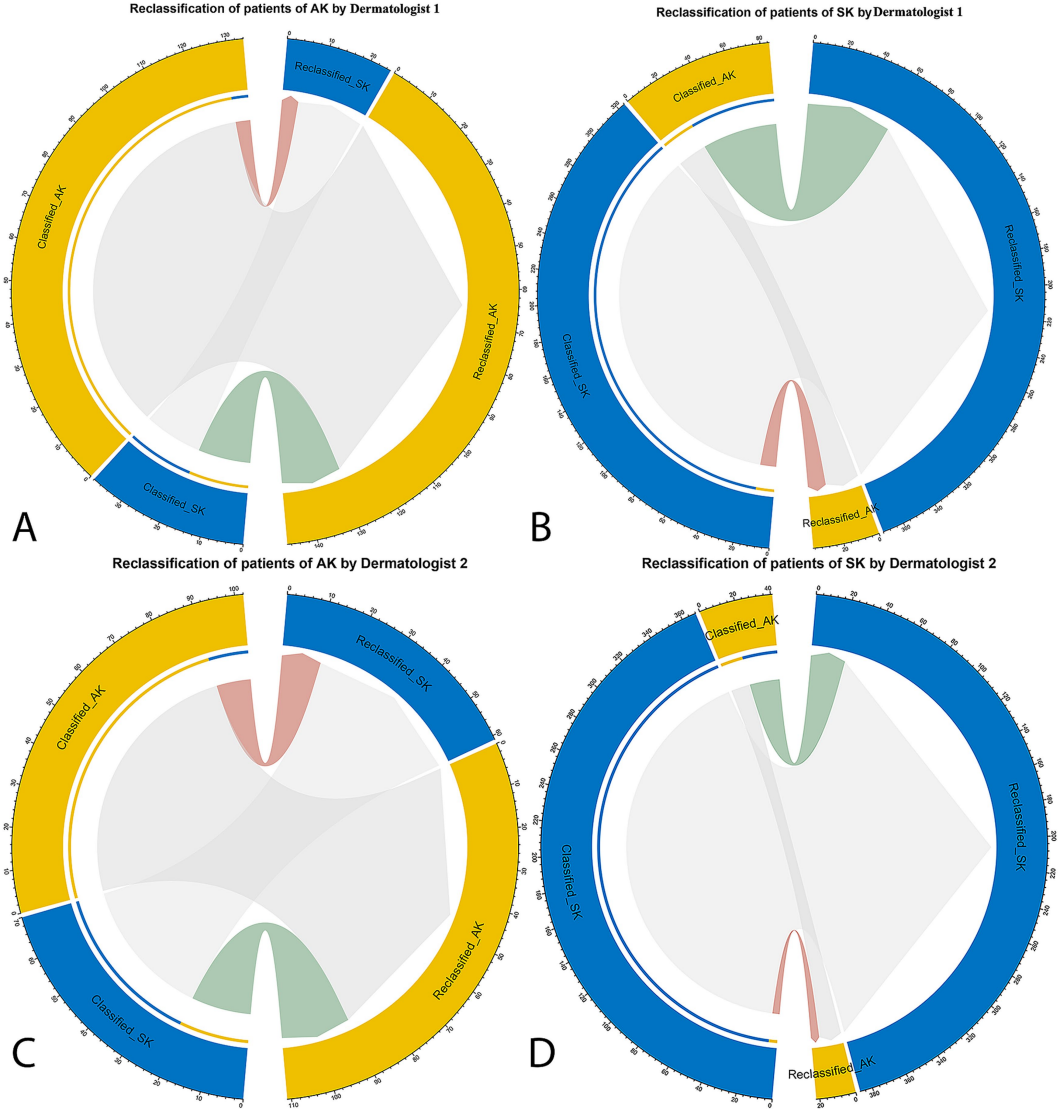
The net reclassification improvement (NRI) analysis for the DL model’s classification of AK and SK. The NRI quantifies the improvement in classification accuracy when incorporating the model’s predictions compared to dermatologists’ initial assessments. In the circle plots, the connections in red represent patients who were reclassified in the incorrect direction, whereas the connections in green indicate patients who were reclassified in the correct direction with the specific patient numbers. **(A)** Dermatologist 1 in classification and reclassification of AK with the assistance of DL model. **(B)** Dermatologist 1 in classification and reclassification of SK with the assistance of DL model. **(C)** Dermatologist 2 in classification and reclassification of AK with the assistance of DL model. **(D)** Dermatologist 2 in classification and reclassification of SK with the assistance of DL model.

The categorical NRI was 0.10 (*p* = 0.006) and 0.19 (*p* < 0.001) for dermatologist 1 and dermatologist 2, indicating a significant improvement with the DL model’s assistance. The IDI was 0.14 (*p* < 0.001) and 0.27 (*p* < 0.001), confirming statistically significant betterment in discrimination between AK and SK with 14 and 27% improvement for dermatologist 1 and dermatologist 2, respectively (data from a merged data set of Center A–C, Figure 4).

### The interpretability of the model

The results showed that when correctly predicted, the model often relied on the color and shape features of the target objects. For both AK and SK the DL model focus on the background color of the images. Additionally, features of objects within the image such as papules or patches on the surface were also factors considered by the model. Furthermore, the smoothness of the skin surface may also be a factor considered by the model. The surfaces of SK were smooth, whereas that of AK were rough (Supplementary Figure 2).

We found that when predictions were incorrect, model failures fell into the following categories: Instances of color blending, Impact of blurry images, Interference from similar colors, and specific features of small sample sizes (Supplementary Figure 3).

## Discussion

This study developed and validated the capability of DL model to enhance dermatological diagnosis for differentiating AK from SK. With the assistance of the DL model, diagnostic accuracy significantly improved. The results suggest that DL model integration into dermatological practice could enhance diagnostic accuracy, reduce subjectivity, and potentially decrease misdiagnosis rates.

Recent studies underscore the efficacy of DL models in dermatology (18, 19). DL models trained on extensive datasets have demonstrated significant capabilities in classifying various skin cancers with notable accuracy (20, 21). Similarly, research indicates that DL models can achieve a diagnostic performance comparable to or exceeding that of dermatologists, particularly in distinguishing between benign and malignant skin conditions (22).

In the specific context of differentiating AK from SK, recent studies have highlighted the promise of DL algorithms. Previous studies used DL models to classify AK and SK with high accuracy and sensitivity (12). Furthermore, studies have explored the use of combining clinical images with patient metadata or histopathological information, to improve the performance of DL models in skin lesion classification (23). Moreover, studies show that an ability of CLIP to learn rich visual representations from large-scale image datasets in medical imaging tasks (24). Unlike conventional CNNs, which primarily learn local image features, CLIP with a ViT backbone can capture long-range contextual relationships across image patches and align them with semantic features. This is particularly valuable in differentiating AK and SK, where subtle differences in surface texture, border irregularity, and pigmentation may require broader contextual modeling. Moreover, CLIP has demonstrated strong performance in medical image analysis tasks due to its ability to leverage pretraining on large-scale image-text pairs, leading to richer and more transferable feature representations.

An important observation from the attention maps was that the model occasionally focused on regions outside the lesion itself, including background skin or surrounding areas. While this may reflect global contextual learning by the model, it also raises valid concerns about potential reliance on spurious features, such as lighting variations or image framing artifacts. From a clinical perspective, diagnostic decisions rely primarily on lesion-specific characteristics such as border irregularity, color heterogeneity, and surface texture (4, 5). The emphasis on non-lesion areas in some cases could reduce interpretability and cast doubt on the model’s alignment with clinical reasoning. To address this limitation, future research should explore incorporating lesion segmentation or masking strategies to constrain the model’s attention to clinically relevant regions. Approaches such as pre-processing images with automated lesion segmentation algorithms, applying attention regularization techniques, or leveraging multi-task learning frameworks that jointly optimize classification and segmentation could help ensure that the model’s decision-making more closely aligns with dermatologists’ clinical reasoning. Further refinement of model training strategies through lesion segmentation-based masking or attention regularization may be necessary to constrain model focus to medically relevant regions, thereby improving both interpretability and reliability, which are critical for clinical integration.

The reclassification analysis highlights the model’s adaptability, a critical feature for clinical applications (25). The NRI and IDI demonstrated significant improvements for the dermatologists in differentiating AK from SK. This comparison highlights the model’s potential to assist dermatologists in differentiating AK from SK, where visual similarities often lead to misclassification. Interestingly, the model improved diagnostic accuracy for both dermatologists, with a significant enchantment for the less-experienced dermatologist. The intended role of our model is as a decision-support system rather than a stand-alone diagnostic tool. Specifically, it can serve as a ‘second reader’ to provide dermatologists with an additional, objective interpretation that may reduce diagnostic uncertainty. Beyond diagnostic assistance, the model could also be applied as a triage tool, flagging potentially malignant or high-risk lesions for more urgent evaluation. By integrating into clinical workflows in these supportive roles, the model has the potential to enhance efficiency and accuracy without replacing dermatologist expertise.

Some limitations should be acknowledged. One limitation of this study is the restricted scope of the dataset. Future work should include multi-ethnic and international datasets to enhance the model’s robustness and generalizability. Furthermore, our study lacks longitudinal and real-world validation, leaving long-term stability, patient outcome impact, and routine practice integration untested. Future research should conduct prospective, longitudinal evaluations in clinical workflows across institutions to assess the practical value and sustainability of AI-assisted dermatological diagnosis. Expanding dataset diversity will be essential to validate the robustness of the model across broader populations and to ensure its fairness and clinical applicability worldwide.

## Conclusion

The CLIP-based ViT DL model substantially enhanced dermatologists’ ability to distinguish AK from SK, with less experienced dermatologists benefiting more significantly. These results imply that such models can aid dermatologists in real-world settings by minimizing diagnostic subjectivity and enhancing the early identification of precancerous lesions, thereby affirming the model’s potential to improve diagnostic accuracy in dermatology. Ultimately, integrating DL models into dermatological practice holds promise for revolutionizing diagnostic approaches and refining therapeutic strategies.

## Data Availability

The raw data supporting the conclusions of this article will be made available by the authors, without undue reservation.

## References

[1] Nataren N Yamada M Prow T. Molecular skin cancer diagnosis: promise and limitations. J Mol Diagn. (2023) 25:17–35. doi: 10.1016/j.jmoldx.2022.09.008, PMID: 36243291

[2] Wang Z Wang X Shi Y Wu S Ding Y Yao G. Advancements in elucidating the pathogenesis of actinic keratosis: present state and future prospects. Front Med. (2024) 11:1330491. doi: 10.3389/fmed.2024.1330491, PMID: 38566927 PMC10985158

[3] Hafner C Vogt T. Seborrheic keratosis. J Dtsch Dermatol Ges. (2008) 6:664–77. doi: 10.1111/j.1610-0387.2008.06788.x, PMID: 18801147

[4] Hames SC Sinnya S Tan JM Morze C Sahebian A Soyer HP. Automated detection of actinic keratoses in clinical photographs. PLoS One. (2015) 10:e0112447. doi: 10.1371/journal.pone.0112447, PMID: 25615930 PMC4304708

[5] Nasiri S Azhari V Bidari-Zerehpoosh F Asadi-Kani Z Talebi A. The diagnostic value of p63, p16, and p53 immunohistochemistry in distinguishing seborrheic keratosis, actinic keratosis, and Bowen's disease. Dermatol Ther. (2021) 34:e14817. doi: 10.1111/dth.14817, PMID: 33497503

[6] Zou DD Sun YZ Li XJ Wu WJ Xu D He YT. Single-cell sequencing highlights heterogeneity and malignant progression in actinic keratosis and cutaneous squamous cell carcinoma. eLife. (2023) 12:e85270. doi: 10.7554/eLife.85270, PMID: 38099574 PMC10783873

[7] van der Velden BHM Kuijf HJ Gilhuijs KGA Viergever MA. Explainable artificial intelligence (XAI) in deep learning-based medical image analysis. Med Image Anal. (2022) 79:102470. doi: 10.1016/j.media.2022.102470, PMID: 35576821

[8] Mirikharaji Z Abhishek K Bissoto A Barata C Avila S Valle E. A survey on deep learning for skin lesion segmentation. Med Image Anal. (2023) 88:102863. doi: 10.1016/j.media.2023.102863, PMID: 37343323

[9] Esteva A Kuprel B Novoa RA Ko J Swetter SM Blau HM. Dermatologist-level classification of skin cancer with deep neural networks. Nature. (2017) 542:115–8. doi: 10.1038/nature21056, PMID: 28117445 PMC8382232

[10] Wang H Qi Q Sun W Li X Yao C. Classification of clinical skin lesions with double-branch networks. Front Med. (2023) 10:1114362. doi: 10.3389/fmed.2023.1114362, PMID: 37358994 PMC10288876

[11] Yu Z Kaizhi S Jianwen H Guanyu Y Yonggang W. A deep learning-based approach toward differentiating scalp psoriasis and seborrheic dermatitis from dermoscopic images. Front Med. (2022) 9:965423. doi: 10.3389/fmed.2022.965423, PMID: 36405606 PMC9669613

[12] Mei LH Cao MK Li J Ye XG Liu XD Yang G. Deep learning in assisting dermatologists in classifying basal cell carcinoma from seborrheic keratosis. Front Oncol. (2025) 15:1507322. doi: 10.3389/fonc.2025.1507322, PMID: 40342818 PMC12058839

[13] Reddy S Giri D Patel R. Artificial intelligence-based distinction of actinic keratosis and seborrheic keratosis. Cureus. (2024) 16:e58692. doi: 10.7759/cureus.58692, PMID: 38774175 PMC11108590

[14] Hentschel S Kobs K Hotho A. CLIP knows image aesthetics. Front Artif Intell. (2022) 5:976235. doi: 10.3389/frai.2022.976235, PMID: 36504688 PMC9732445

[15] Hong S Wu J Zhu L Chen W. Brain tumor classification in VIT-B/16 based on relative position encoding and residual MLP. PLoS One. (2024) 19:e0298102. doi: 10.1371/journal.pone.0298102, PMID: 38954731 PMC11218980

[16] Connor R Dearle A Claydon B Vadicamo L. Correlations of cross-entropy loss in machine learning. Entropy. (2024) 26:491. doi: 10.3390/e26060491, PMID: 38920500 PMC11203011

[17] Pencina MJ D'Agostino RB Vasan RS. Evaluating the added predictive ability of a new marker: from area under the ROC curve to reclassification and beyond. Stat Med. (2008) 27:157–72. doi: 10.1002/sim.2929, PMID: 17569110

[18] Huang NC Mukundan A Karmakar R Syna S Chang WY Wang HC. Novel snapshot-based hyperspectral conversion for dermatological lesion detection via YOLO object detection models. Bioengineering. (2025) 12:714. doi: 10.3390/bioengineering12070714, PMID: 40722406 PMC12292811

[19] Wei ML Tada M So A Torres R. Artificial intelligence and skin cancer. Front Med. (2024) 11:1331895. doi: 10.3389/fmed.2024.1331895, PMID: 38566925 PMC10985205

[20] Lin TL Mukundan A Karmakar R Avala P Chang WY Wang HC. Hyperspectral imaging for enhanced skin cancer classification using machine learning. Bioengineering. (2025) 12:755. doi: 10.3390/bioengineering12070755, PMID: 40722447 PMC12292285

[21] Azeem M Kiani K Mansouri T Topping N. Skinlesnet: classification of skin lesions and detection of melanoma cancer using a novel multi-layer deep convolutional neural network. Cancers (Basel). (2023) 16:108. doi: 10.3390/cancers16010108, PMID: 38201535 PMC10778045

[22] Huang HY Hsiao YP Mukundan A Tsao YM Chang WY Wang HC. Classification of skin cancer using novel hyperspectral imaging engineering via YOLOv5. J Clin Med. (2023) 12:1134. doi: 10.3390/jcm12031134, PMID: 36769781 PMC9918106

[23] Liang X Li X Li F Jiang J Dong Q Wang W. MedFILIP: medical fine-grained language-image pre-training. IEEE J Biomed Health Inform. (2025) 29:3587–97. doi: 10.1109/JBHI.2025.3528196, PMID: 40030972

[24] Ponzio F Descombes X Ambrosetti D. Improving CNNs classification with pathologist-based expertise: the renal cell carcinoma case study. Sci Rep. (2023) 13:15887. doi: 10.1038/s41598-023-42847-y, PMID: 37741835 PMC10517931

[25] Yan BC Li Y Ma FH Zhang GF Feng F Sun MH. Radiologists with MRI-based radiomics aids to predict the pelvic lymph node metastasis in endometrial cancer: a multicenter study. Eur Radiol. (2021) 31:411–22. doi: 10.1007/s00330-020-07099-8, PMID: 32749583

