# Supplementary figures and images for: Enhancing dermatological diagnosis for differentiating actinic from seborrheic keratosis using deep learning model
Source: Front Med (Lausanne). 2025 Oct 2;12:1654813. doi: 10.3389/fmed.2025.1654813 (PMC12528019; doi:10.3389/fmed.2025.1654813)

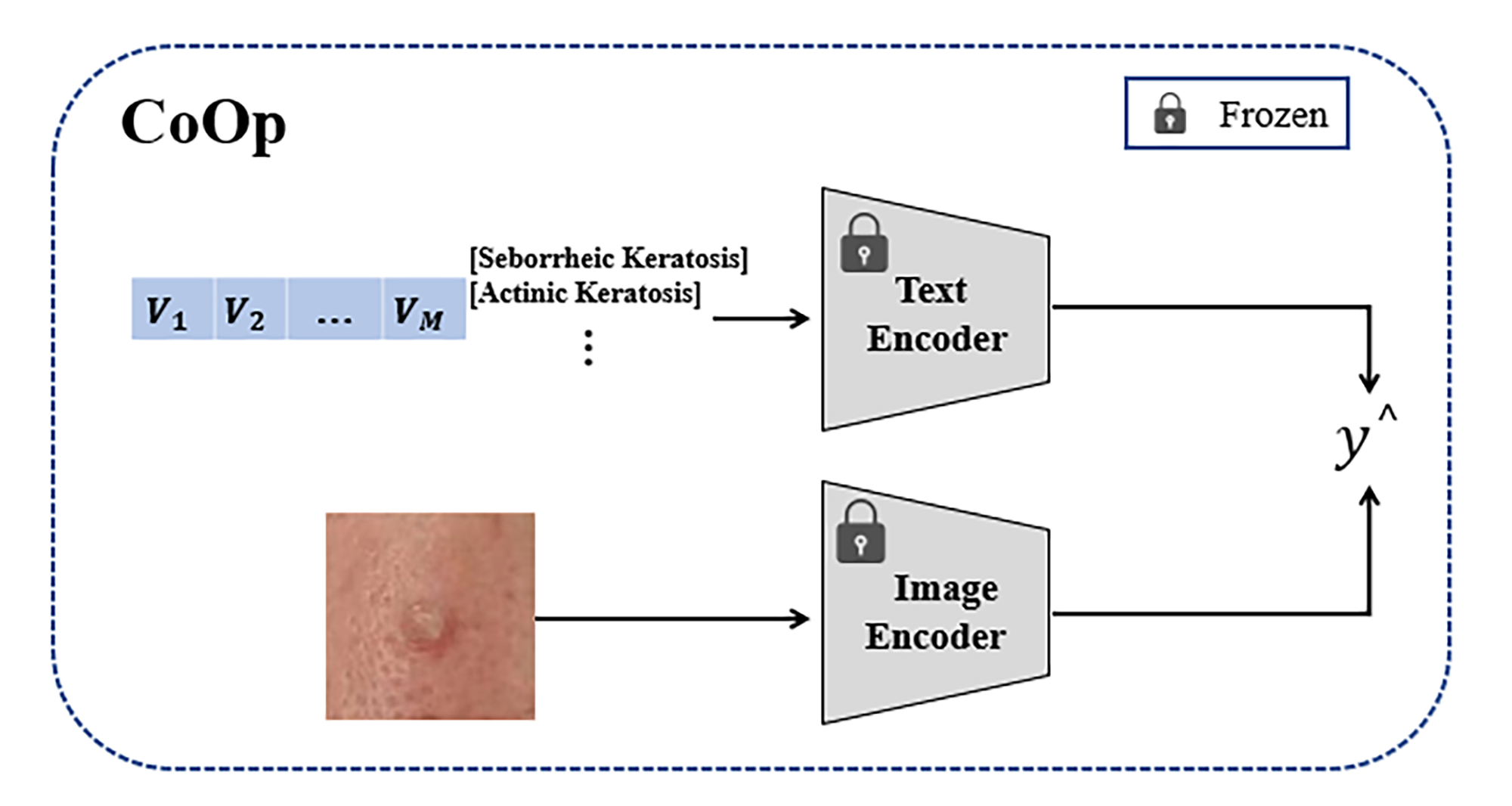

Supplement: Supplementary file 1 [file Image_1.tif]

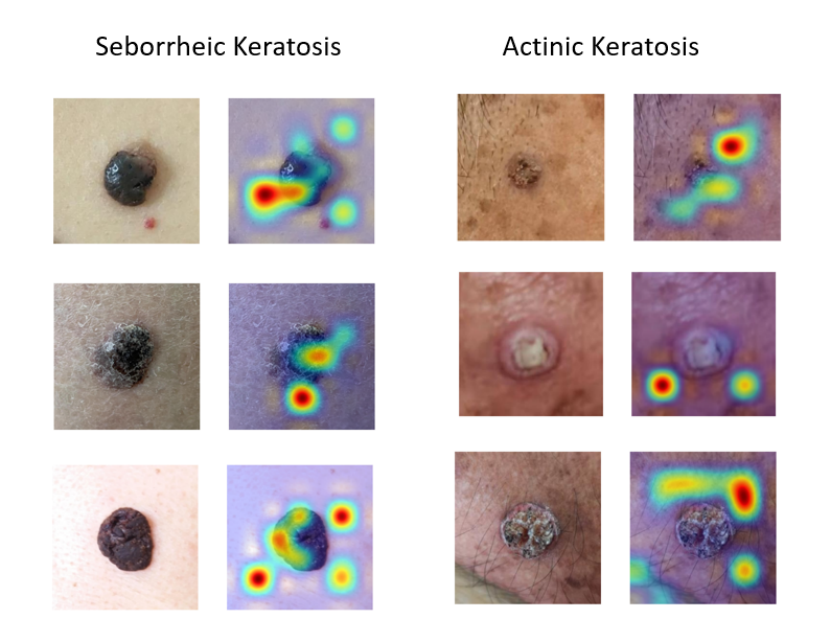

Supplement: Supplementary file 2 [file Image_2.tif]

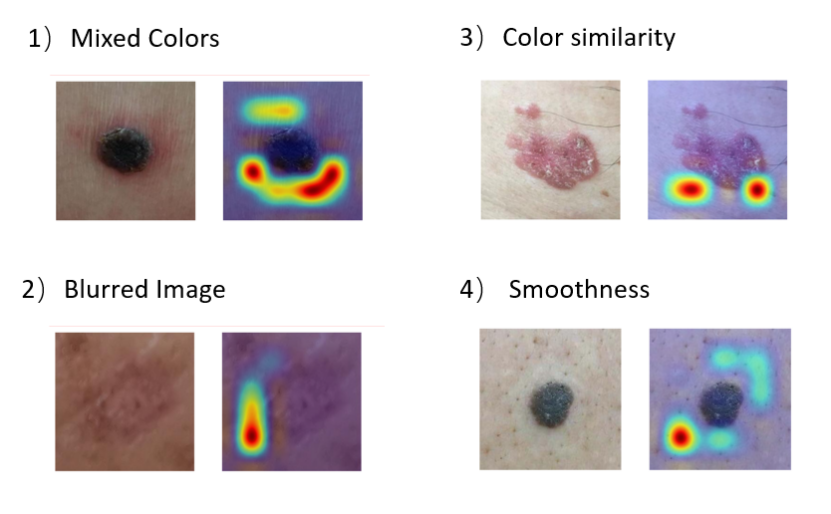

Supplement: Supplementary file 3 [file Image_3.tif]
