# Supplementary material for: Enhancing dermatological diagnosis for differentiating actinic from seborrheic keratosis using deep learning model
Source: Front Med (Lausanne). 2025 Oct 2;12:1654813. doi: 10.3389/fmed.2025.1654813 (PMC12528019; doi:10.3389/fmed.2025.1654813)
Supplement: Supplementary file 4 [file Table_1.docx]

Supplementary Figures

Supplementary Figure 1 The architecture and workflow of the deep learning model used for classifying actinic keratosis (AK) and seborrheic keratosis (SK). The model is based on the CLIP architecture with ViT-B/16. Images are first divided into fixed-size patches, which are then embedded and passed through a Transformer encoder. The encoder captures the relationships between different regions of the image to generate a global representation.

Supplementary Figure 2 The results show that the model's classification of sample categories primarily considered the feature of color. When correctly predicting, the model often relys on the color and shape features of the target objects. Both AK and SK focus on the background color (base) of the images. The color of SK is mostly light brown or brown, while actinic keratosis is mostly light red or dark red. Additionally, features of objects within the image (such as papules or patches on the surface) were also factors considered by the model. SK mostly shows black papillomatous hyperplasia on the surface, while AK often shows light-colored, damaged nodular hyperplasia. Furthermore, the smoothness of the skin surface may also be a factor considered by the model. Most SK surfaces are smooth, whereas actinic keratosis surfaces are rough.

Supplementary Figure 3 The reasons for model prediction failures in samples.

1. Instances of color blending: In certain cases, the color of the affected area may be influenced by surrounding skin or external factors, such as post-scratch erythema. Furthermore, the image not only exhibits the brown color typical of seborrheic dermatitis but also had a superficial red color due to sunlight exposure.
2. Impact of blurry images: Issues of image blurriness caused by improper handling by the photographer or equipment damage can result in model recognition biases regarding color and shape, thus affecting prediction outcomes.
3. Interference from similar colors: For certain skin lesions, the model may be confused due to their colors resembling other lesions.
4. Specific features of small sample sizes: For instance, on originally smooth SK, the presence of enlarged pores due to the patient's condition or other factors may affect the model's prediction results.

Supplementary Table 1 Comparison of the area under the curve (AUC) for model (resnet50 fine-tuned and CLIP) performance on the same data sets by DeLong test

| Models | Data Sets | AUC | 95%CI | SPE | SEN | NPV | PPV | P |
| --- | --- | --- | --- | --- | --- | --- | --- | --- |
| Resnet50 | Training cohort | 0.85 | 0.81-0.89 | 1.00 | 0.7 | 0.89 | 1.00 | 0.108 |
|  | Validation cohort1 | 0.83 | 0.77-0.89 | 0.99 | 0.67 | 0.88 | 0.98 | 0.811 |
|  | Validation cohort2 | 0.85 | 0.83-0.86 | 0.87 | 0.83 | 0.88 | 0.81 | 0.116 |
|  | Validation cohort3 | 0.84 | 0.78-0.89 | 0.88 | 0.79 | 0.56 | 0.96 | 0.103 |
| CLIP | Training cohort | 0.89 | 0.86-0.93 | 0.94 | 0.84 | 0.93 | 0.86 | - |
|  | Validation cohort1 | 0.84 | 0.78-0.9 | 0.98 | 0.71 | 0.89 | 0.93 | - |
|  | Validation cohort2 | 0.85 | 0.84-0.87 | 0.84 | 0.87 | 0.9 | 0.79 | - |
|  | Validation cohort3 | 0.89 | 0.86-0.93 | 0.98 | 0.81 | 0.6 | 0.99 | - |
